# Supplementary material for: Optical circulation in a multimode optomechanical resonator
Source: Nat Commun. 2018 May 4;9:1798. doi: 10.1038/s41467-018-04202-y (PMC5935717; doi:10.1038/s41467-018-04202-y)
Supplement: Supplementary file 1 — Supplementary Information [file 41467_2018_4202_MOESM1_ESM.pdf]

Supplementary Information for "Optical circulation in a multimode optomechanical resonator"

Ruesink et al.

Supplementary Note 1. Experimental setup

Supplementary Figure 1 presents a detailed schematic of the experimental setup used in our experiment. Each port of the device is connected to an output of the optical switch network and to a fast detector (D) via commercially available fibre optic circulators (C, Thorlabs). The electronically controllable optical (SWi) and RF (RFSW) switches, along with the circulators, allow for straightforward measurement of the full transmittance matrix. Four fibre polarisation controllers (FPCs) placed directly after optical switches 2 and 3 ensure that the polarisation of the incoming probe fields can be matched to that of the cavity mode. The polarisation of the control field is separately controlled with a fifth FPC placed directly after the Electro-Optic Modulator (EOM). The FPCs are omitted in the schematic for clarity. We use the EOM for a Pound-Drever-Hall locking scheme that locks the control field to a motional sideband of the optical cavity. Furthermore, two variable optical attenuators (not shown in the schematic) placed after the DPMZI and the EOM control the power levels of the probe and control beams respectively. In our experiments with red- and blue-detuned control fields we used incident control powers of 60 µW and 214 µW, respectively.

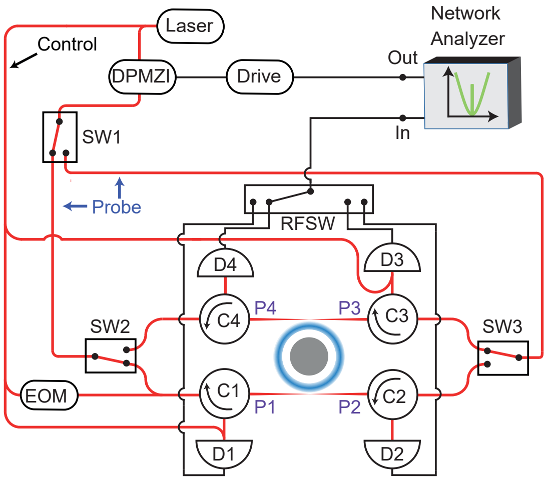

Supplementary Figure 1. **Experimental setup.** A detailed schematic of the experimental setup is shown. The tapered fibres and toroid are placed in a vacuum chamber. DPMZI: double parallel Mach Zehnder interferometer, D1-4: detectors, SW1-3: optical switches, C1-4: circulators, RFSW: radio frequency switch, EOM: electro-optic modulator.

Supplementary Note 2. Measurement and fitting procedure for the transmittance matrix

To provide calibrated and reliable S-matrix elements, the following experimental protocol is followed: first, careful characterization of all the lossy elements between the microtoroid and detectors is performed. This includes all detector responses and losses associated with the commercial components and the tapered fibres. Next, both fibres are gently moved towards the microtoroid until the desired coupling strength is obtained. The latter step involves continuous spectroscopy over a frequency region around ω_c, and is performed at higher probe power levels ($\sim$1 µW) to allow direct measurements of all detector output voltages on an oscilloscope. These voltages are obtained by splitting the detector output lines just before the RFSW (not shown). For the direct channels ($1 \leftrightarrow 2, 3 \leftrightarrow 4$) it is then possible to use the measured off-resonance voltages to normalize the on-resonance cavity response, which directly yields the on-resonance transmittance efficiency.

Considering for example port 1 $\rightarrow$ port 2, the non-resonant voltage on D2, together with the response of the detector and losses of circulator C2 and tapered fibre, allows us to infer the actual power that entered port 1. Using the on-resonance voltages that are recorded by detectors D1/D3/D4 (obtained via fitting a Lorentzian lineshape) and compensating with the appropriate losses and detector response functions, we can then 1) determine the power exiting ports 1/3/4 and 2) calculate the reflection/cross-coupling/add-drop efficiencies. Raw VNA spectra are converted into transmittance spectra with the help of previously determined transmittance efficiencies. This post-processing involves the removal of a small portion of raw VNA spectroscopic data surrounding the mechanical resonance peak. Next, Lorentzian lineshapes are fitted to these cropped (and squared) VNA data, of which the fitted maxima are used to normalize the reflection, cross-coupling and add-drop spectra. For the direct channels, normalization is performed with respect to the minimum of the fitted line. All curves are then multiplied with their respective transmittance efficiencies

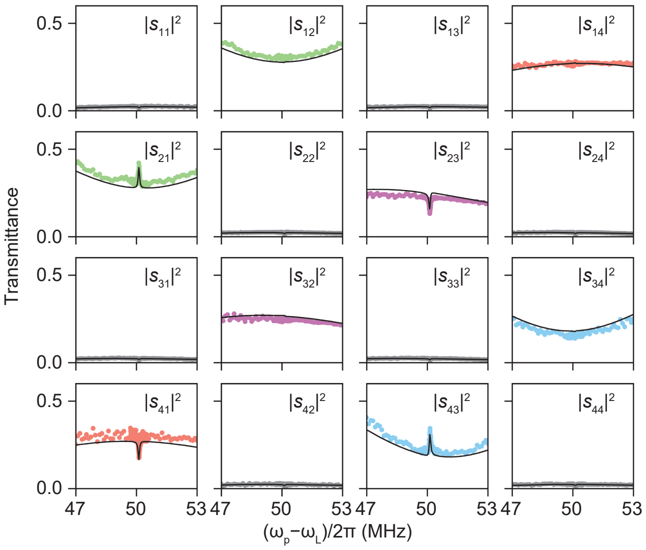

Supplementary Figure 2. **Data for red detuned control.** The 4×4 transmittance matrix spectra that complement the red-detuned control data shown in the manuscript. The lines are global fits to the data set following the procedure described in Supplementary Note 2. The fitting procedure yields $(\kappa_\mathrm{a}, \kappa_\mathrm{b})/2\pi = (2.8, 3.7)$ MHz, mode splitting of $\mu/\pi = 2.8$ MHz and cooperativity of $\mathcal{C} \approx 0.31$. Colour of the data represents the relevant channels of the circulator; data for the forward and reverse directions of a channel are given the same colour.

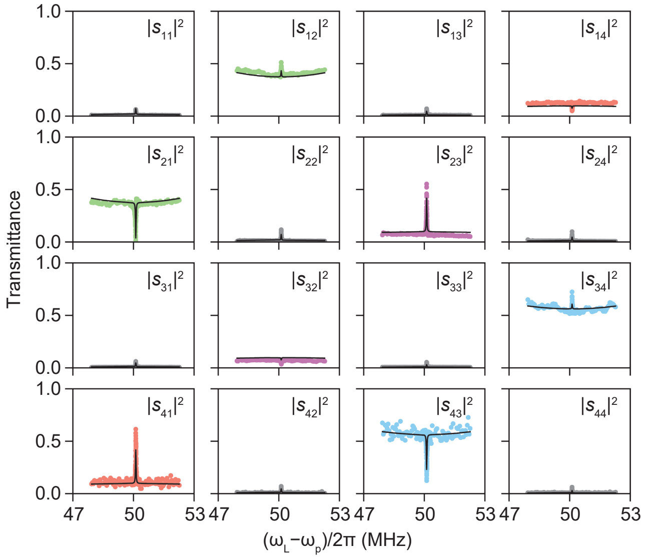

Supplementary Figure 3. **Data for blue detuned control.** The 4×4 transmittance matrix spectra that complement the blue-detuned control data shown in the manuscript along with theoretical fits. The fitting procedure yields $(\kappa_\mathrm{a}, \kappa_\mathrm{b})/2\pi = (2.8, 1.8)$ MHz, mode splitting of $\mu/\pi = 4.3$ MHz and cooperativity of $\mathcal{C} \approx 0.57$. Colour of the data represents the relevant channels of the circulator; data for the forward and reverse directions of a channel are given the same colour.

to yield experimental data. Once the transmittances are obtained, the data around the mechanical resonance peak is included and fitted by varying the mode splitting and cooperativity as fit parameters. During fitting, $\Omega_\mathrm{m}, \Gamma_\mathrm{m}$ and κ_0 are held fixed, as they are obtained from independent spectroscopic measurements. To account for experimental drift during data acquisition, the only parameters that are allowed to vary between the different s-parameters are the control detunings $\bar{\Delta}$. The transmittance spectra for the full 4×4 S matrix, including global fit, are shown in Supplementary Figure 2 (Supplementary Figure 3) for red (blue)-detuned control beam, respectively. The asymmetric transmittance matrices obtained on resonance are shown in Supplementary Figure 4.

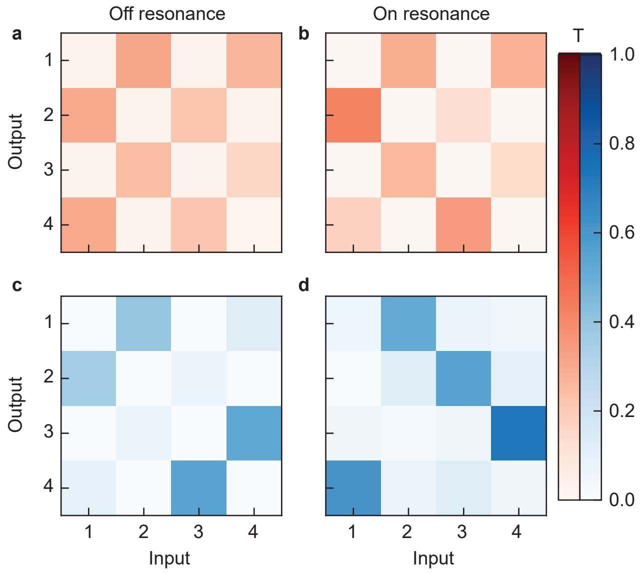

Supplementary Figure 4. **Asymmetric transmittance matrices.** (**a**-**d**) The colourmaps show the transmittance values when the probe detuning frequency ($|\omega_\mathrm{p} - \omega_\mathrm{L}|$) is on (**b**, **d**) and off (**a**, **c**) mechanical resonance. (**a**) and (**b**) correspond to the case of a red detuned control while (**c**) and (**d**) are that of blue detuning. The symmetric transmittance matrix (off-resonance) becomes asymmetric when optomechanical interactions (on-resonance) are present. Especially for the blue detuned data, the performance is close to an ideal circulator.

Supplementary Note 3. Conditions for near-ideal circulation

Here, we examine the scattering matrix of the circulator both in the red- and blue-detuned regimes and investigate the possibility of near-ideal circulation. For simplicity we assume that both the optical modes have equal losses ($\kappa_{\mathrm{a},1} = \kappa_{\mathrm{a},2} = \kappa_\mathrm{a}, \kappa_{\mathrm{b},1} = \kappa_{\mathrm{b},2} = \kappa_\mathrm{b}, \kappa_{0,1} = \kappa_{0,2} = \kappa_0$ and $\kappa = \kappa_0 + \kappa_\mathrm{a} + \kappa_\mathrm{b}$) and are pumped with equal intensity and with $\pi/2$ phase difference ($g_2 = \mathrm{i}g_1 = \mathrm{i}g$). In addition, we focus only at the resonance frequency where the optomechanical intercations are maximal. For a red-detuned system ($\bar{\Delta} = -\Omega_\mathrm{m}$) at resonance ($\omega = \Omega_\mathrm{m}$) the S matrix can be simplified to

$$S = \begin{pmatrix} 0 & 1 & 0 & 0 \\ 1 & 0 & 0 & 0 \\ 0 & 0 & 0 & 1 \\ 0 & 0 & 1 & 0 \end{pmatrix} - \frac{2}{1+\mathcal{C}+\delta^2} \begin{pmatrix} \mathrm{i}\eta_\mathrm{a}\delta & \eta_\mathrm{a}(1+\mathcal{C}) & \mathrm{i}\sqrt{\eta_\mathrm{a}\eta_\mathrm{b}}\delta & \sqrt{\eta_\mathrm{a}\eta_\mathrm{b}}(1+\mathcal{C}) \\ \eta_\mathrm{a} & \mathrm{i}\eta_\mathrm{a}\delta & \sqrt{\eta_\mathrm{a}\eta_\mathrm{b}} & \mathrm{i}\sqrt{\eta_\mathrm{a}\eta_\mathrm{b}}\delta \\ \mathrm{i}\sqrt{\eta_\mathrm{a}\eta_\mathrm{b}}\delta & \sqrt{\eta_\mathrm{a}\eta_\mathrm{b}}(1+\mathcal{C}) & \mathrm{i}\eta_\mathrm{b}\delta & \eta_\mathrm{b}(1+\mathcal{C}) \\ \sqrt{\eta_\mathrm{a}\eta_\mathrm{b}} & \mathrm{i}\sqrt{\eta_\mathrm{a}\eta_\mathrm{b}}\delta & \eta_\mathrm{b} & \mathrm{i}\eta_\mathrm{b}\delta \end{pmatrix} \tag{1}$$

where, $\mathcal{C} = \mathcal{C}_1 + \mathcal{C}_2$ represents the total cooperativity of both modes. In addition, we have defined $\eta_{\mathrm{a,b}} = \frac{\kappa_{\mathrm{a,b}}}{\kappa}$ as the ratio of leakage losses to the total losses of each mode and $\delta = \frac{2\mu}{\kappa}$ as the normalized frequency splitting of the even and odd modes.

Here, the aim is to maximize the forward port-to-port coupling coefficients, i.e., (s_{21}, s_{32}, s_{43}, s_{14}), while minimizing the reverse port-to-port couplings (s_{12}, s_{23}, s_{34}, s_{41}). On the other hand, of interest would be to simultaneously minimize the reflection coefficients ($s_{11}, s_{22}, s_{33}, s_{44}$) and the cross-coupling terms ($s_{13}, s_{31}, s_{24}, s_{42}$). According to Supplementary Equation 1, ideal circulation can be acheieved in the limit of large cooperativities ($\mathcal{C} \to \infty$) and for zero internal losses when assuming equal mode coupling to the upper and lower waveguide channels ($\eta_{\mathrm{a,b}} \to 1/2$). On the other hand, another interesting observation in the S matrix of Supplementary Equation 1 is that the reflection coefficients and the cross-coupling terms are all proportional with the frequency splitting 2μ (appearing as δ in the S matrix). This can be understood easily in the basis of rotating whispering gallery modes. In such picture, any coupling between the counter-rotating modes is mediated through their mutual coupling rate μ which instead results in a finite reflection and coupling of the diagonal ports.

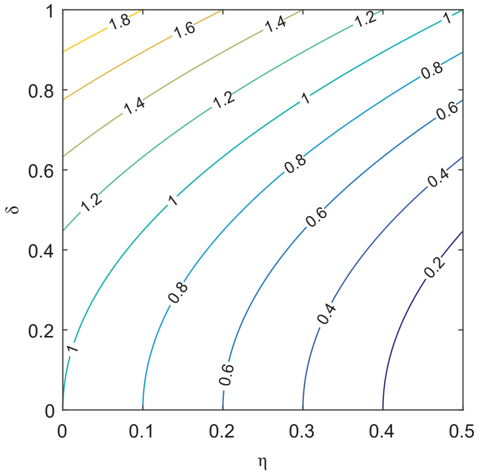

Supplementary Figure 5. **Optimal circulation.** The required cooperativity for optimal circulation in the blue-detuned regime in a parameter map of η and δ.

Similarly, for a blue-detuned control ($\bar{\Delta} = \Omega_{\mathrm{m}}$) at resonance ($\omega = -\Omega_{\mathrm{m}}$) the S matrix can be written as

$$S = \begin{pmatrix} 0 & 1 & 0 & 0 \\ 1 & 0 & 0 & 0 \\ 0 & 0 & 0 & 1 \\ 0 & 0 & 1 & 0 \end{pmatrix} - \frac{2}{1 - \mathcal{C} + \delta^2} \begin{pmatrix} \mathrm{i}\eta_{\mathrm{a}}\delta & \eta_{\mathrm{a}}(1-\mathcal{C}) & \mathrm{i}\sqrt{\eta_{\mathrm{a}}\eta_{\mathrm{b}}}\delta & \sqrt{\eta_{\mathrm{a}}\eta_{\mathrm{b}}}(1-\mathcal{C}) \\ \eta_{\mathrm{a}} & \mathrm{i}\eta_{\mathrm{a}}\delta & \sqrt{\eta_{\mathrm{a}}\eta_{\mathrm{b}}} & \mathrm{i}\sqrt{\eta_{\mathrm{a}}\eta_{\mathrm{b}}} \\ \mathrm{i}\sqrt{\eta_{\mathrm{a}}\eta_{\mathrm{b}}}\delta & \sqrt{\eta_{\mathrm{a}}\eta_{\mathrm{b}}}(1-\mathcal{C}) & \mathrm{i}\eta_{\mathrm{b}}\delta & \eta_{\mathrm{b}}(1-\mathcal{C}) \\ \sqrt{\eta_{\mathrm{a}}\eta_{\mathrm{b}}} & \mathrm{i}\sqrt{\eta_{\mathrm{a}}\eta_{\mathrm{b}}}\delta & \eta_{\mathrm{b}} & \mathrm{i}\eta_{\mathrm{b}}\delta \end{pmatrix}. \tag{2}$$

which is similar to that of the red-detuned system when replacing $\mathcal{C}$ with $-\mathcal{C}$. In a similar fashion, one can show that in this case a large cooperativity results in ideal circulation, however, this is an unphysical scenario given that in the blue-detuned regime large cooperativities give rise to parametric instabilities. On the other hand, the S matrix of Supplementary Equation 2 exhibits interesting properties and can become close to that of an ideal circulator for critical choices of the parameters involved. Interestingly, compared to the red-detuned case, here the circulation direction is reversed, i.e., $(s_{12}, s_{23}, s_{34}, s_{41})$ can be close to unity while $(s_{21}, s_{32}, s_{43}, s_{14})$ are minimum. In fact, for a particular set of parameters, one can show that s_{21} and s_{43} become zero for

$$\mathcal{C} = 1 + \delta^2 - 2\eta_{\mathrm{a}} \tag{3}$$

$$\mathcal{C} = 1 + \delta^2 - 2\eta_{\mathrm{b}} \tag{4}$$

which clearly requires equal leakage rate for both channels $\eta_{\mathrm{a,b}} = \eta$. In addition, these conditions demand a total cooperativity larger than the ratio of internal loss to total losses ($\eta_0 = 1 - 2\eta$). Supplementary Figure 5 depicts the cooperativity required to satisfy this condition for different values of η and δ. Interestingly, the choice of $\delta^2 = 2\eta$ demands a cooperativity of $\mathcal{C} = 1$ which instead results in the following resonant S matrix:

$$S = \begin{pmatrix} -\mathrm{i}\delta & 1 & -\mathrm{i}\delta & 0 \\ 0 & -\mathrm{i}\delta & -1 & -\mathrm{i}\delta \\ -\mathrm{i}\delta & 0 & -\mathrm{i}\delta & 1 \\ -1 & -\mathrm{i}\delta & 0 & -\mathrm{i}\delta \end{pmatrix} \tag{5}$$

According to this simple relation, near-ideal circulation can be achieved for $\mathcal{C} = 1$ and $\delta^2 = 2\eta$. On the other hand, one can reduce the reflection coefficients and diagonal port couplings by decreasing δ which instead requires a decrease in η. This latter scenario thus happens for a weak waveguide-cavity coupling.

Supplementary Note 4. Parametric instability threshold

For a blue-detuned control laser, the optomechanical system can enter a parametric instability regime. For a single-mode optomechanical system, the threshold cooperativity associated with the onset of instabilities is found to be

$\mathcal{C}_{\rm th}=1$. Similar relation holds for the microtoroid system when assuming a degenerate pair of counter rotating modes where only one of the modes is populated by the control laser. However, assuming a finite coupling μ between the two modes, this relation is no longer valid. In this case, one would expect a higher cooperativity for bringing the system to instability threshold given that the active mode is coupled to another mode which is naturally lossy. For a blue-detuned system, the dynamical equations governing the optomechanical system in the absence of input probe signals can be written as:

$$\frac{d}{dt}\begin{pmatrix}\delta a_1\\ b^\dagger\\ \delta a_2\end{pmatrix}=\mathrm{i}\begin{pmatrix}\bar\Delta_1+\mathrm{i}\kappa_1/2 & g_1 & 0\\ -g_1^* & \Omega_{\rm m}+\mathrm{i}\Gamma_{\rm m}/2 & -g_2^*\\ 0 & g_2 & \bar\Delta_2+\mathrm{i}\kappa_2/2\end{pmatrix}\begin{pmatrix}\delta a_1\\ b^\dagger\\ \delta a_2\end{pmatrix} \tag{6}$$

Assuming $\bar\Delta_{1,2}=\Omega_{\rm m}\pm\mu$ and $g_2=\mathrm{i}g_1=\mathrm{i}g$, we consider an ansatz of $\left(\delta a_1\ ,\ b^\dagger\ ,\ \delta a_2\right)^T=\left(\alpha_1\ ,\ \beta\ ,\ \alpha_2\right)^{\rm T}e^{i\Omega_{\rm m}t}e^{st}$, which results in the following cubic equation for the exponent s

$$s^3+\left(\kappa+\frac{\Gamma_{\rm m}}{2}\right)s^2+\left(\mu^2+\frac{\kappa^2}{4}+\kappa\frac{\Gamma_{\rm m}}{2}-2|g|^2\right)s+\mu^2\frac{\Gamma_{\rm m}}{2}+\frac{\kappa^2}{4}\frac{\Gamma_{\rm m}}{2}-\kappa|g|^2=0. \tag{7}$$

The stability of the system can now be investigated by finding a parameter regime for which all roots of this equation fall on the left side of the imaginary axis, i.e., Real$(s)<0$. This latter can be obtained through the Routh-Hurwitz stability criterion. According to the Routh-Hurwitz criterion, a generic cubic equation of the form $s^3+a_2s^2+a_1s+a_0$ has all roots in the negative real part plane as long as $a_0>0$, $a_2>0$ and $a_1a_2>a_0$. For Supplementary Equation 7, the stability conditions are found to be:

$$\frac{\Gamma_{\rm m}}{2}\left(\mu^2+\frac{\kappa^2}{4}\right)>\kappa|g|^2, \tag{8}$$

$$\left(\kappa+\frac{\Gamma_{\rm m}}{2}\right)\left(\mu^2+\frac{\kappa^2}{4}+\kappa\frac{\Gamma_{\rm m}}{2}-2|g|^2\right)>\frac{\Gamma_{\rm m}}{2}\left(\mu^2+\frac{\kappa^2}{4}\right)-\kappa|g|^2, \tag{9}$$

Given that in practice $\Gamma_{\rm m}\ll\kappa$, the first condition imposes a lower bound on the control power required to bring the system to instability threshold. Therefore, by rewriting Supplementary Equation 8 in terms of the total cooperativity, the condition of stability can be written as $\mathcal{C}<1+\delta^2$, which results into the following expression for the instability threshold

$$\mathcal{C}_{\rm th}=1+\delta^2. \tag{10}$$

This relation assures that the condition of near-ideal circulation (Supplementary Equation 3) is accessible without running into instability. It is worth noting that this condition is obtained under the rotating wave approximation. A more general expression can be found when considering both mechanical sidebands.

Supplementary Note 5. Circulation bandwidth

According to the results presented in the main text, the bandwidth of the optomechanical circulator is dictated by the linewidth of the optomechanically induced transparency (OMIT) or absorption (OMIA) window. Considering the S-matrix derived in the Materials and Methods section, the bandwidth can be obtained through the frequency-dependent factor of $[\det(\omega I+M)]^{-1}$,

$$\frac{1}{\det(\omega I+M)}=\frac{1}{\Sigma_{\rm o}^2(\omega)-\mu^2\mp2|g|^2\Sigma_{\rm o}(\omega)/\Sigma_{\rm m}(\omega)}, \tag{11}$$

where, to simplify the analysis, we have assumed equal losses in both modes, $\kappa_1=\kappa_2$, and equal intensity pumping, $|g_1|=|g_2|$. In this relation the up and down signs are associated with the red- and blue-detuned regimes respectively. Given that in general $\kappa\gg\Gamma_{\rm m}$, the inverse optical susceptibilities are fairly constant near the resonance $\omega=\pm\Omega_{\rm m}$ and over the small frequency range of OMIT/OMIA. Thus one can assume $\Sigma_{\rm o}(\omega)\approx\mathrm{i}\kappa/2$ which greatly simplifies

Supplementary Equation 11 to

$$\frac{1}{\det(\omega I + M)} = -\frac{4}{\kappa^2(1+\delta^2)}\left(1 - \mathrm{i}\frac{\pm\frac{\mathcal{C}}{1+\delta^2}}{\omega \mp \Omega_\mathrm{m} + \mathrm{i}\left(1 \pm \frac{\mathcal{C}}{1+\delta^2}\right)\frac{\Gamma_\mathrm{m}}{2}}\right). \tag{12}$$

According to this relation, the transparency or absorption window is found to be

$$\mathrm{BW} = \left(1 \pm \frac{\mathcal{C}}{1+\delta^2}\right)\Gamma_\mathrm{m}, \tag{13}$$

which, could be compared with a similar relation for a single-mode optomechanical cavity: $\mathrm{BW} = (1 \pm \mathcal{C})\Gamma_\mathrm{m}$. Recalling that $\delta = \mu/(\kappa/2)$ is a normalized mode splitting, Supplementary Equation 13 shows that in general the lifted degeneracy of the modes decreases the bandwidth in the red-detuned regime while it increases the bandwidth in the blue-detuned case. In addition, Supplementary Equation 13 is in agreement with the fact that the bandwidth should approach zero at the onset of parametric instabilities.

Supplementary Note 6. Noise analysis

Thermal phonons in the mechanical resonator interact with the control beam creating cavity photons that contribute to noise at the output ports of the device. Together with the vacuum noise the total noise to the outputs at each port for the cases of red and blue detuned controls is discussed below.

Noise contribution for red detuned control

The effect of noise can be considered in the linearized equation of motion as:

$$\frac{d}{dt}\begin{pmatrix}\delta a_1\\ \delta a_2\\ b\end{pmatrix} = \mathrm{i}M_\mathrm{red}\begin{pmatrix}\delta a_1\\ \delta a_2\\ b\end{pmatrix} + D^\mathrm{T}\begin{pmatrix}\delta s_1^+\\ \delta s_2^+\\ \delta s_3^+\\ \delta s_4^+\\ \sigma_{1,\mathrm{in}}\\ \sigma_{2,\mathrm{in}}\\ b_\mathrm{in}\end{pmatrix} \tag{14}$$

where $\sigma_{1,\mathrm{in}}$, $\sigma_{2,\mathrm{in}}$, and b_in correspond to the fluctuations entering the optical modes and mechanical mode, respectively, from the baths that are associated with the intrinsic loss channels. The fluctuations δs_j^+ enter from the four optical input waveguide ports.

Here the matrices M_red and D^T correspond to

$$M_\mathrm{red} = \begin{pmatrix}\mu + \mathrm{i}\kappa_1/2 & 0 & g_1\\ 0 & -\mu + \mathrm{i}\kappa_2/2 & g_2\\ g_1^* & g_2^* & \mathrm{i}\Gamma_\mathrm{m}/2\end{pmatrix} \tag{15}$$

and

$$D^\mathrm{T} = \frac{1}{\sqrt{2}}\begin{pmatrix}\mathrm{i}\sqrt{\kappa_{\mathrm{a},1}} & \mathrm{i}\sqrt{\kappa_{\mathrm{a},1}} & \mathrm{i}\sqrt{\kappa_{\mathrm{b},1}} & \mathrm{i}\sqrt{\kappa_{\mathrm{b},1}} & \sqrt{2\kappa_{0,1}} & 0 & 0\\ -\sqrt{\kappa_{\mathrm{a},2}} & \sqrt{\kappa_{\mathrm{a},2}} & -\sqrt{\kappa_{\mathrm{b},2}} & \sqrt{\kappa_{\mathrm{b},2}} & 0 & \sqrt{2\kappa_{0,2}} & 0\\ 0 & 0 & 0 & 0 & 0 & 0 & \sqrt{2\Gamma_\mathrm{m}}\end{pmatrix}. \tag{16}$$

The output at each port can then be written as

$$\begin{pmatrix} \delta s_1^- \\ \delta s_2^- \\ \delta s_3^- \\ \delta s_4^- \\ \sigma_{1,\mathrm{out}} \\ \sigma_{2,\mathrm{out}} \\ b_{\mathrm{out}} \end{pmatrix} = C \begin{pmatrix} \delta s_1^+ \\ \delta s_2^+ \\ \delta s_3^+ \\ \delta s_4^+ \\ \sigma_{1,\mathrm{in}} \\ \sigma_{2,\mathrm{in}} \\ b_{\mathrm{in}} \end{pmatrix} + D \begin{pmatrix} \delta a_1 \\ \delta a_2 \\ b \end{pmatrix} \tag{17}$$

where $\sigma_{1,\mathrm{out}}$, $\sigma_{2,\mathrm{out}}$, and b_{out} are the corresponding dissipation ports and

$$C = \begin{pmatrix} 0 & 1 & 0 & 0 & 0 & 0 & 0 \\ 1 & 0 & 0 & 0 & 0 & 0 & 0 \\ 0 & 0 & 0 & 1 & 0 & 0 & 0 \\ 0 & 0 & 1 & 0 & 0 & 0 & 0 \\ 0 & 0 & 0 & 0 & 1 & 0 & 0 \\ 0 & 0 & 0 & 0 & 0 & 1 & 0 \\ 0 & 0 & 0 & 0 & 0 & 0 & 1 \end{pmatrix}. \tag{18}$$

We assume that the noise operators obey correlations given by:

$$\left\langle \delta s_j^+(t) \delta s_j^{+\dagger}(t') \right\rangle = \delta(t-t') \tag{19}$$

$$\left\langle \sigma_{1,\mathrm{in}}(t) \delta\sigma_{1,\mathrm{in}}^\dagger(t') \right\rangle = \delta(t-t') \tag{20}$$

$$\left\langle \sigma_{2,\mathrm{in}}(t) \delta\sigma_{2,\mathrm{in}}^\dagger(t') \right\rangle = \delta(t-t') \tag{21}$$

$$\left\langle b_{\mathrm{in}}(t) b_{\mathrm{in}}^\dagger(t') \right\rangle = (\bar{n}_{\mathrm{th}} + 1)\delta(t-t') \tag{22}$$

where $j = 1 - 4$ and the optical bath is approximated to be at zero temperature.

Combining the above equations we get

$$\begin{pmatrix} \delta s_1^- \\ \delta s_2^- \\ \delta s_3^- \\ \delta s_4^- \\ \sigma_{1,\mathrm{out}} \\ \sigma_{2,\mathrm{out}} \\ b_{\mathrm{out}} \end{pmatrix} (\omega) = S(\omega) \begin{pmatrix} \delta s_1^+ \\ \delta s_2^+ \\ \delta s_3^+ \\ \delta s_4^+ \\ \sigma_{1,\mathrm{in}} \\ \sigma_{2,\mathrm{in}} \\ b_{\mathrm{in}} \end{pmatrix} \tag{23}$$

with

$$S(\omega) = C + \mathrm{i}D(\omega I + M_{\mathrm{red}})^{-1} D^{\mathrm{T}} \tag{24}$$

where the elements $S_{1:4,1:4}$ form the scattering matrix presented in the main text.

The symmetrized noise power spectral density at the four output ports of the waveguides is then given by [1]

$$\bar{S}_j^-(\omega) = \frac{1}{2} \int_{-\infty}^{\infty} dt e^{\mathrm{i}\omega t} \left\langle \delta s_j^{-\dagger}(t) \delta s_j^-(0) + \delta s_j^-(0) \delta s_j^{-\dagger}(t) \right\rangle \tag{25}$$

for $j = 1 - 4$, which leads to

$$\begin{aligned} \bar{S}_j^-(\omega) = & \frac{1}{2} \left|S_{j1}(-\omega)\right|^2 + \frac{1}{2} \left|S_{j2}(-\omega)\right|^2 + \frac{1}{2} \left|S_{j3}(-\omega)\right|^2 + \frac{1}{2} \left|S_{j4}(-\omega)\right|^2 \\ & + \frac{1}{2} \left|S_{j5}(-\omega)\right|^2 + \frac{1}{2} \left|S_{j6}(-\omega)\right|^2 + \left(\bar{n}_{\mathrm{th}} + \frac{1}{2}\right) \left|S_{j7}(-\omega)\right|^2 . \end{aligned} \tag{26}$$

We consider the S matrix only at resonance ($\omega = 0$) and under the following assumptions:

$$g_2 = \mathrm{i}g_1 = \mathrm{i}g \tag{27}$$

$$\kappa_1 = \kappa_2 = \kappa \tag{28}$$

$$\kappa_{0,1} = \kappa_{0,2} = (1-2\eta)\kappa \tag{29}$$

$$\kappa_{a,1} = \kappa_{b,1} = \kappa_{a,2} = \kappa_{b,2} = \eta\kappa \tag{30}$$

such that the S matrix, written in terms of the cooperativity and normalized mode splitting, becomes

$$S = \frac{1}{1+\mathcal{C}+\delta^2}\begin{pmatrix} -\mathrm{i}2\eta\delta & \delta^2 & -\mathrm{i}2\eta\delta & -2\eta(1+\mathcal{C}) & 0 & 0 & -\mathrm{i}2\delta\sqrt{\eta\mathcal{C}} \\ \delta^2+\mathcal{C} & -\mathrm{i}2\eta\delta & -2\eta & -\mathrm{i}2\eta\delta & 0 & 0 & -2\sqrt{\eta\mathcal{C}} \\ -\mathrm{i}2\eta\delta & -2\eta(1+\mathcal{C}) & -\mathrm{i}2\eta\delta & \delta^2 & 0 & 0 & -\mathrm{i}2\delta\sqrt{\eta\mathcal{C}} \\ -2\eta & -\mathrm{i}2\eta\delta & \mathcal{C}+\delta^2 & -\mathrm{i}2\eta\delta & 0 & 0 & -2\sqrt{\eta\mathcal{C}} \\ 0 & 0 & 0 & 0 & 1+\mathcal{C}+\delta^2 & 0 & 0 \\ 0 & 0 & 0 & 0 & 0 & 1+\mathcal{C}+\delta^2 & 0 \\ -2\sqrt{\eta\mathcal{C}} & -\mathrm{i}2\delta\sqrt{\eta\mathcal{C}} & -2\sqrt{\eta\mathcal{C}} & -\mathrm{i}2\delta\sqrt{\eta\mathcal{C}} & 0 & 0 & 3+\mathcal{C}+3\delta^2 \end{pmatrix}. \tag{31}$$

For the optimal condition of $\eta = \frac{1}{2}$, Supplementary Equation 26 and Supplementary Equation 31 can be used to simplify the output noise spectral density at each port as

$$N_{1,3} = \bar{S}_{1,3}(0) = \frac{1}{2} + \frac{2\mathcal{C}\delta^2}{(1+\mathcal{C}+\delta^2)^2}\bar{n}_{\mathrm{th}} \tag{32}$$

$$N_{2,4} = \bar{S}_{2,4}(0) = \frac{1}{2} + \frac{2\mathcal{C}}{(1+\mathcal{C}+\delta^2)^2}\bar{n}_{\mathrm{th}}. \tag{33}$$

The noise contribution arising from a non-zero thermal population, $\bar{n}_{\mathrm{th}}$, is seen to reduce in the limit of $\mathcal{C} \to \infty$ and in the limit of zero thermal population of the mechanical resonator the output noise is seen to be limited only by vacuum noise. The added noise to port j, referred back to the input port k, can then be calculated considering the channel transmittance and the input signal contribution as $n_{\mathrm{add},j} = N_j/|S_{jk}|^2 - \frac{1}{2}$. In the red detuned regime, we consider the transmittances $|S_{21}|^2$, $|S_{32}|^2$, $|S_{43}|^2$, and $|S_{14}|^2$, where the added noise is then given by

$$n_{\mathrm{add},1,3} = \frac{\delta^2(\delta^2+2(1+\mathcal{C})+4\mathcal{C}\bar{n}_{\mathrm{th}})}{2(1+\mathcal{C})^2} \tag{34}$$

$$n_{\mathrm{add},2,4} = \frac{1+2\mathcal{C}+2\delta^2+4\mathcal{C}\bar{n}_{\mathrm{th}}}{2(\mathcal{C}+\delta^2)^2}. \tag{35}$$

For degenerate optical modes ($\delta = 0$) the added noise to ports 1 and 3 (for a signal inserted at ports 4 and 2 respectively) is seen to be independent of thermal and vacuum noise.

Noise contribution for blue detuned control

A similar analysis can be followed for blue-detuned control. Then Supplementary Equations 14-24 can be used when replacing all b, b_{in}, b_{out} by their Hermitian conjugates, and replacing M_{red} by

$$M_{\mathrm{blue}} = \begin{pmatrix} \mu+\mathrm{i}\kappa_1/2 & 0 & g_1 \\ 0 & -\mu+\mathrm{i}\kappa_2/2 & g_2 \\ -g_1^* & -g_2^* & \mathrm{i}\Gamma_{\mathrm{m}}/2 \end{pmatrix}. \tag{36}$$

We consider the contribution only on resonance ($\omega = 0$) and use the same assumptions on g_i and κ_i as earlier. The noise power spectral density has the same form as in Supplementary Equation 25. Considering optimal conditions, the output noise for each port for the blue detuned case is given by

$$N_{1,3} = \frac{1}{2} + 2(\bar{n}_{\mathrm{th}}+1) \tag{37}$$

$$N_{2,4} = \frac{1}{2} + \frac{2}{\delta^2}(\bar{n}_{\mathrm{th}}+1) \tag{38}$$

where we have assumed $\mathcal{C} = 1$ and $\delta^2 = 2\eta$. The noise in each port, for these conditions, is seen to be decided by the tolerance for reflections and cross-couplings, which is equal to δ^2. In this regime, as the transmittances for the relevant channels are unity, the added noise becomes $N_j - \frac{1}{2}$ and is given by

$$n_{\text{add},1,3} = 2(\bar{n}_{\text{th}} + 1) \tag{39}$$

$$n_{\text{add},2,4} = \frac{2}{\delta^2}(\bar{n}_{\text{th}} + 1). \tag{40}$$

Supplementary References

[1] Clerk, A. A., Devoret, M. H., Girvin, S. M., Marquardt, F. & Schoelkopf, R. J. Introduction to quantum noise, measurement, and amplification. *Rev. Mod. Phys.* **82**, 1155 (2010).
